# A case of shock after 10 days systemic corticosteroid therapy for COVID-19

**DOI:** 10.1186/s40981-020-00410-y

**Published:** 2021-01-08

**Authors:** Keiko Uemura, Satoki Inoue, Masahiko Kawaguchi

**Affiliations:** Department of Anesthesiology and Division of Intensive Care, Nara Medical University, 840 Shijo-cho Kashihara, Nara, 634-8522 Japan

To the Editor,

Systemic corticosteroids are recommended for the treatment of lung injury in patients with severe and critical coronavirus disease 2019 (COVID-19) [1, 2]. We encountered a case of shock after the completion of systemic corticosteroid therapy for COVID-19.

A 79-year-old male patient with hypertension was admitted to the intensive care unit (ICU) with lung injury related to the severe acute respiratory syndrome coronavirus 2 (SARS-CoV-2) infection. Hypertension was treated with amlodipine 5 mg/day. But, his physical status had been good, and he had been attending the gym three times per week until he became sick. Immediately after admission, his trachea was intubated and his lungs were mechanically ventilated (PEEP = 12 cmH_2_O, driving pressure = 13 cmH_2_O, inspiratory time = 1.5 s). Prone positioning combined with muscular relaxation for 48 h was applied. Dexamethasone 6.6 mg/day and remdesivir 100 mg for 10 days were also administered. During prone positioning, his oxygenation improved; however, his oxygenation deteriorated again in the supine position (Fig. 1). Two days after the completion of prone positioning therapy, he was suspected to develop ventilator-associated pneumonia (VAP), which was treated with cefepime 3 g/day (Fig. 2a). As time elapsed, his oxygenation did not show much changes, although his chest X-ray image appeared to improve (Fig. 1). Despite the suspicion of pulmonary embolism, the level of fibrin degradation product (FDP) was low, and no findings of right ventricular dysfunction were observed. After the completion of 10 days of dexamethasone therapy, steroid administration was stopped without tapering or replacement. At 20 h after the cessation of dexamethasone, his blood pressure slightly decreased, which required an increment of noradrenaline infusion and fluid infusion. Over the next 12 h, we observed the deterioration of hemodynamic status and hypoglycemia (Fig. 1). Embolic complication was again ruled out with low FDP and unimpaired right ventricular function. Chest X-ray revealed right dominant pulmonary edema (Fig. 2b). Hydrocortisone 100 mg, followed by continuous infusion of 200 mg/day, was administered, after which his hemodynamics and glucose levels became stable. High PEEP (15 cmH_2_O) provided a transient improvement in the oxygenation and chest X-ray image. However, he died due to progressive respiratory failure (Fig. 1).

**Fig. 1:**
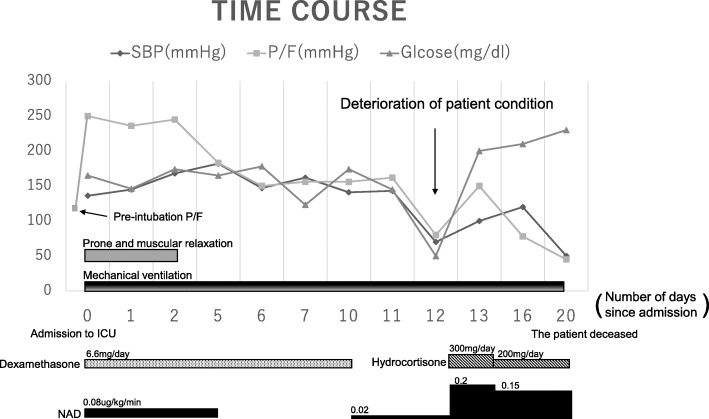
The clinical course. SBP, systolic blood pressure (mmHg); P/F, the ratio of arterial oxygen partial pressure to fractional inspired oxygen (mmHg); Glucose, blood sugar (mg/dl); NAD, noradrenaline (μg/kg/min). The units of the vertical axis mean mmHg for SBP and P/F, or md/dl for glucose. The unit of the horizontal axis means the number of days since ICU admission. The stepped graphs show the doses of continuous infusion of NAD and steroids. Belt-lines show the periods of mechanical ventilation and prone positioning combined with muscle relaxation. His pre-intubation P/F ratio was approximately 110 mmHg

**Fig. 2:**
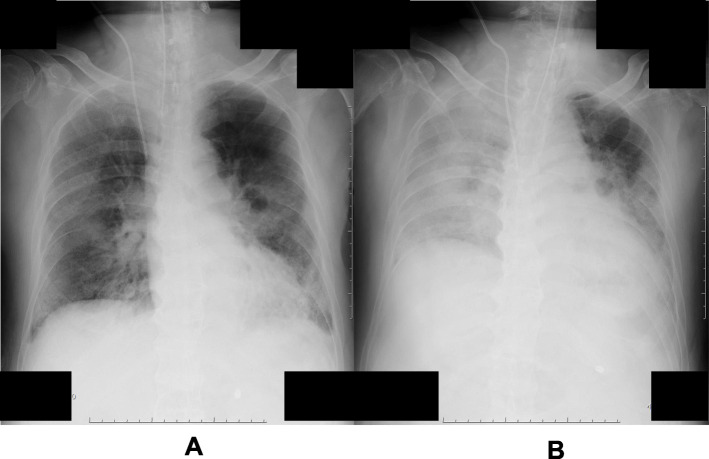
**a** Suspected ventilator-associated pneumonia (VAP). Two days after completion of prone positioning therapy (4 days after starting administration of dexamethasone), he was suspected to develop VAP, which was prominent in the right middle to the lower lobes. His P/F was around 150 mmHg at that time. **b** Unilateral pulmonary edema was observed in the right lung. Thirty-six hours after cessation of dexamethasone, he developed unilateral pulmonary edema. His P/F was deteriorated down to 80 mmHg

It has been suggested that synthetic steroids can induce a persistent inhibition of the hypothalamic–pituitary–adrenal (HPA) axis, even after a short period of treatment, which is concerned about patients with COVID-19 infection who are treated with corticosteroids [3]. Moreover, clinical guidelines for critical illness-related corticosteroid insufficiency recommend that corticosteroid therapy should not be stopped abruptly because deterioration may occur due to the development of a reconstituted inflammatory response, especially in acute respiratory distress syndrome [4]. In the present case, it is reasonable to believe that the abrupt cessation of corticosteroids induced adrenal insufficiency because there was a dramatic improvement in the exacerbation of his systemic condition with the administration of hydrocortisone. Considering that the half-life of dexamethasone is 36–72 h [2], it almost satisfied the onset of a series of symptoms. Therefore, the development of a reconstituted inflammatory response might have caused the right dominant pulmonary edema. However, we did not evaluate the serum cortisol or adrenocorticotropic hormone (ACTH) levels of the patient, which is a major limitation of this case report.

In conclusion, it is important to consider that 10-day dexamethasone administration might inhibit the adrenal response to stress in some patients with COVID-19 infection, even in those who were previously healthy, when dexamethasone is recognized as a routine and first-line therapy for severe to critical COVID-19 infection.

## Data Availability

Not applicable.

## Abbreviations

COVID-19: Coronavirus disease 2019
SARS-CoV-2: Severe acute respiratory syndrome coronavirus 2
ICU: Intensive care unit
P/F: Ratio of arterial oxygen partial pressure to fractional inspired oxygen
FDP: Fibrin degradation product
VAP: Ventilator-associated pneumonia
PEEP: Positive end-expiratory pressure
HPA: Hypothalamic–pituitary–adrenal
ACTH: Adrenocorticotropic hormone
